# Altered functional connectivity between primary motor cortex subregions and the whole brain in patients with incomplete cervical spinal cord injury

**DOI:** 10.3389/fnins.2022.996325

**Published:** 2022-11-03

**Authors:** Ling Wang, Weimin Zheng, Beining Yang, Qian Chen, Xuejing Li, Xin Chen, Yongsheng Hu, Lei Cao, Jian Ren, Wen Qin, Yanhui Yang, Jie Lu, Nan Chen

**Affiliations:** ^1^Department of Radiology, Xuanwu Hospital, Capital Medical University, Beijing, China; ^2^Beijing Key Laboratory of Magnetic Resonance Imaging and Brain Informatics, Beijing, China; ^3^Department of Radiology, Beijing Friendship Hospital, Capital Medical University, Beijing, China; ^4^Department of Radiology, China Rehabilitation Research Center, Beijing, China; ^5^Department of Functional Neurosurgery, Xuanwu Hospital, Capital Medical University, Beijing, China; ^6^Department of Rehabilitation Medicine, Xuanwu Hospital, Capital Medical University, Beijing, China; ^7^Department of Neurosurgery, Xuanwu Hospital, Capital Medical University, Beijing, China; ^8^Department of Radiology, Tianjin Medical University General Hospital, Beijing, China

**Keywords:** incomplete cervical spinal cord injury, primary motor cortex, subregion, gray matter volume, functional connectivity

## Abstract

To investigate the reorganizations of gray matter volume (GMV) in each subregion of primary motor cortex (M1) after incomplete cervical cord injury (ICCI) and to explore the differences in functional connectivity (FC) between the M1 subregions and the whole brain, and further to disclose the potential value of each M1 subregion in motor function rehabilitation of ICCI patients. Eighteen ICCI patients and eighteen age- and gender- matched healthy controls (HCs) were recruited in this study. The 3D high-resolution T1-weighted structural images and resting-state functional magnetic resonance imaging (rs-fMRI) of all subjects were obtained using a 3.0 Tesla MRI system. Based on the Human Brainnetome Atlas, the structural and functional changes of M1 subregions (including A4hf, A6cdl, A4ul, A4t, A4tl, A6cvl) in ICCI patients were analyzed by voxel-based morphometry (VBM) and seed-based FC, respectively. Compared with HCs, no structural changes in the M1 subregions of ICCI patients was detected. However, when compared with HCs, ICCI patients exhibited decreased FC in visual related areas (lingual gyrus, fusiform gyrus) and sensorimotor related areas (primary sensorimotor cortex) when the seeds were located in bilateral A4hf, A4ul, and decreased FC in visual related areas (lingual gyrus, fusiform gyrus) and cognitive related areas (temporal pole) when the seed was located in the left A4t. Moreover, when the seeds were located in the bilateral A6cdl, decreased FC in visual related areas (lingual gyrus, fusiform gyrus, calcarine gyrus) was also observed. Our findings demonstrated that each of the M1 regions had diverse FC reorganizations, which may provide a theoretical basis for the selection of precise stimulation targets, such as transcranial magnetic stimulation (TMS) or transcranial direct current stimulation (tCDS), meanwhile, our results may reveal the possible mechanism of visual feedback and cognitive training to promote motor rehabilitation.

## Introduction

The primary motor cortex (M1) works together with other brain regions to coordinate the voluntary movement of the whole body (Mohammed and Hollis, 2018). It contains Betz cells, which are the large neurons that communicate with alpha motor neurons through the axons of the spinal cord (Szocsics et al., 2021). Theoretically, Wallerian degeneration (WD) occurred and spread along the corticospinal tract due to the partial or complete interruption following spinal cord injury (SCI) (Buss et al., 2004), resulting in the structural and functional alterations of M1 (Freund et al., 2013; Athanasiou et al., 2017; Nardone et al., 2018; Li et al., 2020). The reorganization of M1 may be the key factor affecting the motor function rehabilitation of SCI patients (Hou et al., 2014b; Athanasiou et al., 2017). However, the issue of whether SCI leads to brain cortex reorganization in M1 remains controversial. Therefore, clarifying the structural and functional changes of M1 is helpful to reveal the mechanism of motor rehabilitation and its related treatments.

At present, some scholars found that the gray matter volume (GMV) of M1 would be decreased (Freund et al., 2011, 2013; Hou et al., 2014b; Seif et al., 2018) due to the apoptosis of axons (Pallini et al., 1988), the neuron atrophy (Wannier et al., 2005), and the reduction in dendritic spine density (Kim et al., 2006), while some others failed to find GMV changes in M1 after SCI (Yoon et al., 2013; Villiger et al., 2015; Chen et al., 2017; Pan et al., 2017; Guo et al., 2021). Extensive collateral connections within M1 and connections between M1 and other higher-order motor areas may maintain cell activity in the motor system, thereby preventing the volume atrophy in M1 after SCI (Chen et al., 2017; Nardone et al., 2018).

Apart from the inconsistent changes in GMV, the functional reorganization of M1 after SCI, especially the changes of functional connectivity (FC), is also controversial. Some studies (Min et al., 2015; Rao et al., 2016) have demonstrated that the FC between M1 and other motor areas was significantly increased to compensate for the motor deficits after SCI (Min et al., 2015; Athanasiou et al., 2017). However, some others have shown decreased FC between M1 and other motor areas (Oni-Orisan et al., 2016; Pan et al., 2017), which points to possible decreased interhemispheric communication in the resting state (Oni-Orisan et al., 2016). Moreover, some scholars even found decreased interhemispheric FC between the bilateral M1, and increased FC within the motor network in each individual hemisphere, including M1, premotor cortex, supplementary motor area, thalamus, and cerebellum. And these alterations may influence the prognosis of SCI patients (Hou et al., 2014a; Athanasiou et al., 2017). In addition to the alterations of FC within the motor network, some scholars have found that M1 became less connected with the visual cortex after SCI (Hawasli et al., 2017; Li et al., 2020), while others did not find any changes of FC between M1 and visual related brain regions (Oni-Orisan et al., 2016).

The contradictory results may be partly due to the different research methods or different research objects, such as the difference of injury type, injury degree, injury level, or duration time. Nevertheless, it is more likely due to that previous studies on SCI patients have researched the changes of M1 based on the whole brain or the whole M1 as region of interest (ROI) (Yoon et al., 2013; Villiger et al., 2015; Chen et al., 2017; Pan et al., 2017; Guo et al., 2021). In fact, M1 contains a wide range of regions and complex internal functions, and is composed of multiple subregions (Fan et al., 2016). Each subregion of M1 has different functions (Fan et al., 2016). This may be the reason for the inconsistent results of previous studies. Some studies based on M1 subregions have been carried out in patients with Parkinson’s syndrome (Shen et al., 2020), stroke (Liu et al., 2022), and autism spectrum disorder (Nebel et al., 2014), providing basis and new insights for clinical intervention. However, the reorganizations of M1 subregions after SCI have not been reported. In recent years, neural regulation technologies, such as transcranial magnetic stimulation (TMS) and transcranial direct current stimulation (tCDS), have been used in the motor rehabilitation of SCI patients (Zheng et al., 2020). However, the stimulation to different parts of M1 may result in different effects (Tazoe and Perez, 2015). Therefore, the study of the structural and functional changes of M1 subregions in SCI patients will not only help to reveal the neural mechanism of motor rehabilitation, but also provide more accurate treatment targets for neural regulation.

Based on previous studies, we hypothesized that the changes of GMV and/or FC in each M1 subregion may be varied, leading to different results in the whole M1. And the diverse reorganizations in each M1 subregion may lead to different effects of the neural regulation technologies (TMS/r tDCS). Therefore, based on the M1 subregions of the Human Brainnetome Atlas (Fan et al., 2016), we will explore the structural changes in the whole or each subregion of M1 using voxel-based morphometry (VBM), and investigate the functional alterations through analyzing the FC between M1 subregions and all voxels of the whole brain, aiming to clarify the structural and functional characteristics of M1 subregions in SCI patients.

## Materials and methods

### Participants

The current study was approved by the Ethics Committee of Xuanwu Hospital of Capital Medical University and was in accordance with the Declaration of Helsinki. We obtained informed written consents from each adult subject and from the guardians of minor subjects before participating in this study. In addition to a physical examination, all participants underwent a neurological examination as well. Except for sensorimotor disorders, no other dysfunction was found in all patients, and they all met the inclusion criteria: traumatic incomplete cervical cord injury (ICCI), duration time more than 1 weeks, without the history of associated brain diseases confirmed by conventional magnetic resonance imaging (MRI), no pre-existing mental illness or cognitive disorders. Healthy controls (HCs) were selected according to the following criteria: the age and gender were roughly matched with that of the ICCI group, and all of the HCs had no signs or symptoms of neurological disorders, or a history of drug or alcohol abuse, head trauma, or mental illness. Thus, a total of 36 right-handed participants were recruited for this study, including 18 ICCI patients (10 males and 8 females, with a mean age of 50.33 ± 14.62 years and an age range of 16–71 years) and 18 HCs (10 male and 8 female controls with a mean age of 52.78 ± 9.03 years and a range of 27–65 years). In ICCI group, except three patients were classified as grade C, others are grade D according to the American Spinal Injury Association (ASIA) Impairment Scale 2012.^1^ Detailed information of ICCI patients is shown in Table 1.

**TABLE 1:** Clinical data of ICCI patients.

Subjects	Age (years)	Gender	Etiology	Duration (weeks)	Level of lesion	Side of the injury	ASIA	Motor (0–100)	Sensory (0–224)
1	55	F	Fall	39	C3-4	Left	D	80	188
2	51	M	Fall	4	C3-4	Bilateral	D	92	220
3	65	F	Accident	9	C4-6	Bilateral	D	70	176
4	22	M	Fall	1	C6	Bilateral	D	100	216
5	71	M	Surgery	53	C3-4	Bilateral	D	80	204
6	53	M	Trauma	2	C5-6	Bilateral	D	90	204
7	47	M	Accident	1	C5	Bilateral	D	94	208
8	48	M	Fall	2	C3-7	Bilateral	C	33	128
9	36	M	Fall	2	C6-7	Bilateral	D	40	186
10	58	M	Fall	1	C3-5	Bilateral	D	62	216
11	46	F	Accident	1	C2-4	Bilateral	D	76	200
12	56	M	Fall	1	C4-6	Bilateral	D	80	224
13	56	M	Fall	5	C3-7	Bilateral	C	34	132
14	70	F	Fall	6	C1-2	Bilateral	D	100	216
15	62	F	Accident	1	C3-4	Bilateral	D	60	112
16	53	F	Accident	1	C5-7	Bilateral	D	90	212
17	41	F	Trauma	39	C4-5	Bilateral	D	85	116
18	16	F	Accident	53	C5-7	Bilateral	C	24	62

The level of lesion refers to the neurological level. ICCI, incomplete cervical cord injury; ASIA impairment scale: A: complete—no sensory or motor function is preserved in sacral segments S4–S5; B, incomplete—sensory but not motor function is preserved below the neurological level and extends through sacral segments S4–S5; C, incomplete—motor function is preserved below the neurological level, and more than half of the key muscles below the neurological level have a muscle grade of < 3; D, incomplete—motor function is preserved below the neurological level, and at least half of the key muscles below the neurological level have a muscle grade of > 3; Sensory score, sum of segmental light touch and pinprick classifications, ASIA, American Spinal Injury Association.

### Magnetic resonance imaging data acquisition

Magnetic resonance imaging data were collected using a 3.0 T MRI system (Siemens, Trio Tim, Erlangen, Germany) and a 12-channel phase-array head coil. Conventional brain axial fluid-attenuated inversion recovery (FLAIR) sequence was obtained to exclude visible brain abnormalities. The high-resolution 3D structural T1-weighted images were acquired by a three-dimensional (3D) magnetization- prepared rapid gradient-echo sequence (MPRAGE) in sagittal orientation. The imaging parameters were as follows: repetition time (TR) = 1800 ms, echo time (TE) = 2.13 ms, inversion time (TI) = 1100 ms, flip angle (FA) = 9°, field of view (FOV) = 256 mm × 256 mm, matrix size = 256 × 256, number of slices = 192, slice thickness = 1 mm, resulting an isotropic voxel size of 1 mm × 1 mm × 1 mm. Participants were instructed to stay awake, relax, and close their eyes during the resting-state data acquisition, then a gradient-echo-planar imaging (EPI) pulse sequence was used to collect the resting-state functional MRI (rs-fMRI) data, and the parameters were as follows: TR = 2000 ms, TE = 30 ms, slice thickness = 3 mm, inter-slice gap 1 mm, number of slices = 35, FOV = 220 mm × 220 mm, matrix size = 64 × 64, FA = 9°. The parameters resulted in an anisotropic voxel size of 3.4 mm × 3.4 mm × 3.0 mm. The total acquisition time of the resting-state fMRI scan was 6.08 min with 180 volumes.

### Definition of primary motor cortex subregions

**FIGURE 1:**
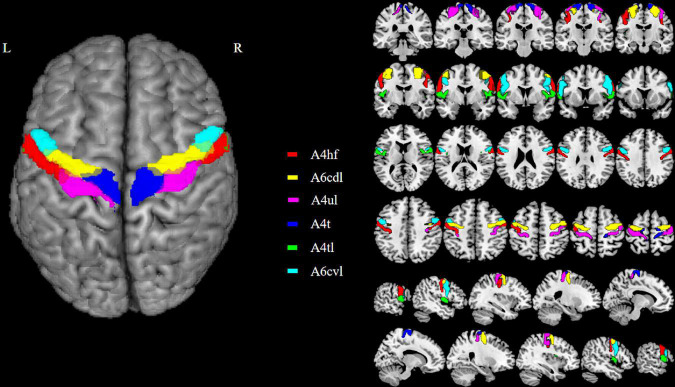
The top viewport displays the subregions of M1. There are 12 M1 subregions in the Human Brainnetome Atlas, including bilateral A4hf, A6cdl, A4ul, A4t, A4tl, and A6cvl. M1, primary motor cortex.

The M1 subregions were defined according to the Human Brainnetome Atlas which is based on the connectional architecture and applies multimodal neuroimaging techniques (Fan et al., 2016). In each hemisphere, M1 was divided into six subregions: PrG-1 [area 4 (head and face region); A4hf], PrG-2 (caudal dorsolateral area 6; A6cdl), PrG-3 [area 4 (upper limb region); A4ul], PrG-4 [area 4 (trunk region); A4t], PrG-5 [area 4 (tongue and larynx region); A4tl], PrG-6 (caudal ventrolateral area 6; A6cvl). The bilateral M1 thus has 12 ROIs in total. Detailed information on the ROIs is provided in Figure 1 and Table 2.

**TABLE 2:** Primary motor cortex subregions within the Brainnetome atlas.

Left and right hemispheres	Modified cyto-architectonic	lh.MNI (X, Y, Z)	rh.MNI (X, Y, Z)
PrG_L(R)_6_1	A4hf, area 4 (head and face region)	–49, –8, 39	55, –2, 33
PrG_L(R)_6_2	A6cdl, caudal dorsolateral area 6	–32, –9, 58	33, –7, 57
PrG_L(R)_6_3	A4ul, area 4 (upper limb region)	–26, –25, 63	34, –19, 59
PrG_L(R)_6_4	A4t, area 4 (trunk region)	–13, –20, 73	15, –22, 71
PrG_L(R)_6_5	A4tl, area 4 (tongue and larynx region)	–52, 0, 8	54, 4, 9
PrG_L(R)_6_6	A6cvl, caudal ventrolateral area 6	–49, 5, 30	51, 7, 30

M1, primary motor cortex; MNI, Montreal Neurological Institute; lh, left hemisphere; rh, right hemisphere; PrG, precentral gyrus; L, left; R, right.

### Image data preprocessing and analyses

#### Structural data preprocessing

All the raw DICOM images were checked and then converted to Neuroimaging Informatics Technology Initiative format using the dcm2nii routine of MRICRON software.^2^ The next step was to perform the preprocessing steps using the CAT12 toolboxes with the default settings described in detail in the CAT12 manual.^3^ The 3D T1-weighted MRI scans were normalized using an affine followed by non-linear registration, corrected for bias field in homogeneities, and then segmented into gray matter (GM), white matter (WM), and cerebrospinal fluid (CSF) components. After segmentation, the segmented scans were normalized into a standard Montreal Neurological Institute (MNI) space using the Diffeomorphic Anatomical Registration Through Exponentiated Lie (DARTEL) algebra algorithm. The normalized GM component was modulated to generate the relative GMV by multiplied by the non-linear part of the deformation field at the DARTEL step. Then, in order to improve the signal-to-noise ratio, meet the statistical requirements of Gaussian random field theory and compensate for residual anatomic variations, the normalized GM images were smoothed with a Gaussian kernel of 8 mm full width at half maximum (FWHM).

#### Functional image processing

The rs-fMRI data preprocessing was performed using the Data Processing & Analysis for Brain Imaging (DPABI).^4^ The first 10 volumes of the time series were discarded to ensure signal stabilization. Then, the remaining images were slice-timing corrected, realigned, and resliced for head motion correction. Rigid body registration was used to evaluate and correct head motion between volumes, and the fMRI data of all subjects were within defined head motion thresholds (maximum translation or rotation less than 3.0 mm or 3.0°). Subsequently, the corrected images were spatially normalized to the standard stereotactic space defined by the MNI template and each voxel was then resampled to an isotropic size of 3 mm × 3 mm × 3 mm. As a next step, the fMRI images were smoothed using a 6 mm FWHM, and to remove possible variances from the time course of each voxel, 26 nuisance covariates, including WM and CSF signals, as well as Friston 24 head motion parameters, were regressed. After that, linear detrending and band-pass filtering (0.01–0.08 Hz) were carried out to control low-frequency drift and high-frequency physiological noise. Then, the M1 subregions were defined as seeds, and connectivity networks at the individual level were obtained using a seed-based approach. The mean time series of each M1 seed were extracted from each participant’s preprocessed functional images. And correlation analysis was conducted between the voxels in seed region and the remaining voxels within the entire brain. For improving the Gaussianity of their distribution, the resulting *r* values were converted to z maps using Fisher’s r-to-z transformation. Group-averaged FC maps for each seed were calculated for each group.

#### Statistical analyses

The mean GMV values in each M1 subregion and the whole M1 region of all the individuals were extracted using DPABI, and all of them were normally distributed checked by Shapiro–Wilk test. Two sample *t*-tests were then used to compare the GMV in each ROI between the two groups on SPSS version 22.0 (IBM, Armonk, NY, USA). The significance level was set at *P* < 0.05/14 = 0.0036 (14 comparisons for 12 M1 subregions and 2 whole M1 regions) with the Bonferroni correction method.

Second-level analyses for each seed-based FC map were conducted using SPM12 (Statistical Parametric Mapping).^5^ One sample *t*-tests were performed to explore the brain regions which are functionally connected to the M1 seeds of both groups [family wise error (FWE) corrected *P* < 0.05, cluster size > 100 voxels]. Then, in order to explore the FC differences of the M1 subregions between ICCI patients and HCs, two-sample *t*-tests was performed with age and gender as covariates. The significance threshold was set to cluster-level FWE correction (*P* < 0.05, two-tailed).

Finally, in order to determine the possible correlation between GMV and FC values in brain regions with significant group differences and clinical variables (including sensory scores, motor scores, and injury duration), spearman correlation analyses were performed with age and gender as covariates (*P* < 0.05).

## Results

### Demographic and clinical characteristics

No statistically significant differences were found between ICCI patients and HCs in age (*t*-test, *T* = 0.603, *P* = 0.550) and gender (Chi-square test, *P* = 1.000).

### Morphological changes of primary motor cortex in incomplete cervical cord injury patients

There was no significant GMV difference in the whole or each subregion of M1 between ICCI patients and the HCs.

### Altered functional connectivity of primary motor cortex subregions

**FIGURE 2:**
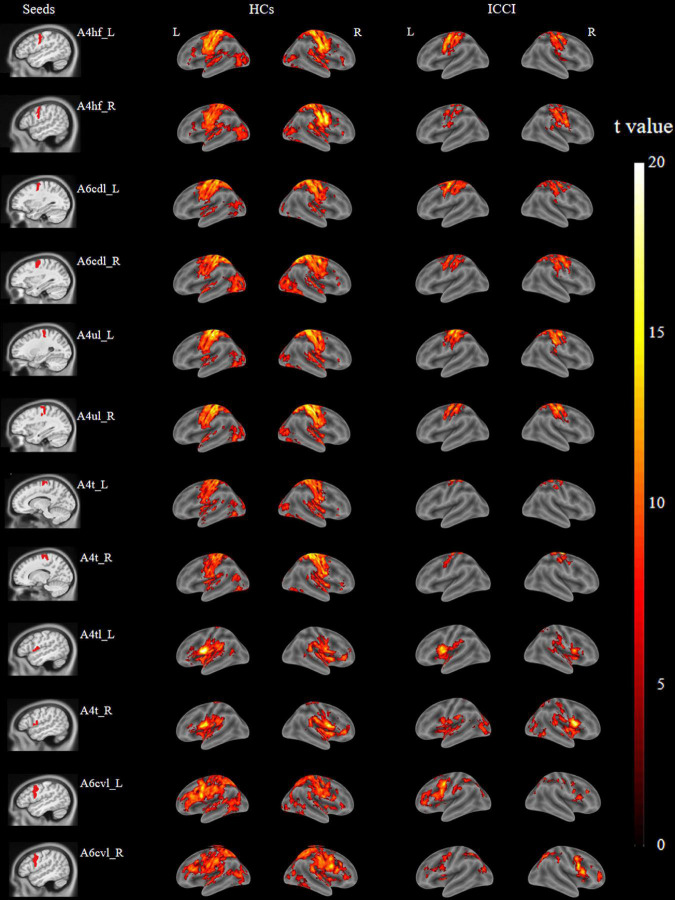
Brain regions functionally connected to M1 subregions of HCs and ICCI patients. In this figure, displayed the typical brain regions functionally connected to M1 subregions of both groups [family wise error (FWE) corrected *P* < 0.05 and cluster size > 100 voxels]. M1, primary motor cortex; HCs, healthy controls; ICCI, incomplete cervical cord injury.

**FIGURE 3:**
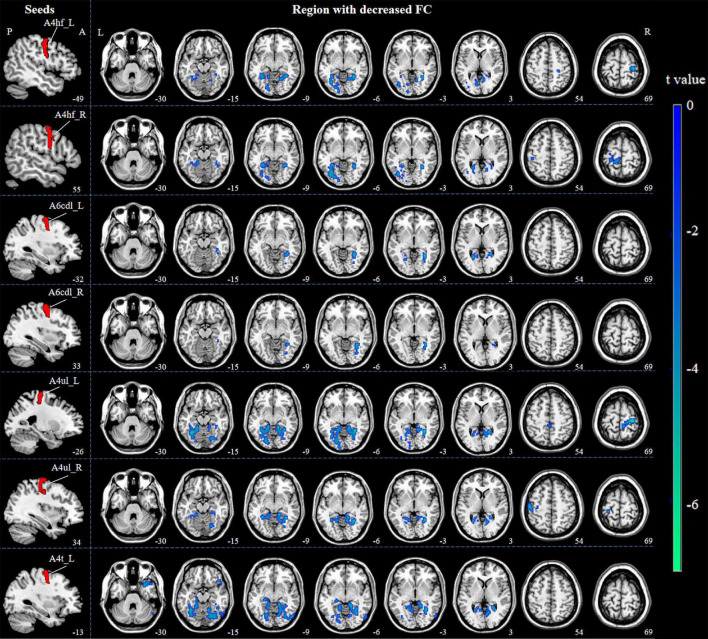
Decreased FC between M1 subregions and the whole brain in ICCI patients. Compared with HCs, ICCI patients demonstrated decreased FC in bilateral LG, left FG and right PSMC when the seed was located in the left A4hf, as well as decreased FC in bilateral FG and left PSMC when the seed was located in the right A4hf. Moreover, ICCI patients showed decreased FC between the left A6cdl and right LG, left CG, as well as decreased FC between the right A6cdl and right FG. In addition, decreased FC between the left A4ul and bilateral FG, right PSMC as well as decreased FC between the right A4ul and left LG, PSMC, right FG was also found in ICCI patients. Besides, when the seed was located in the left A4t, ICCI patients demonstrated decreased FC in right TP, LG and left FG [corrected at cluster level with family wise error (FWE) *P* < 0.05 and cluster size > 75]. FC, functional connectivity; M1, primary motor cortex; ICCI, incomplete cervical cord injury; HCs, healthy controls; LG, lingual gyrus; FG, fusiform gyrus; PSMC, primary sensorimotor cortex; CG, calcarine gyrus; TP, temporal pole.

The brain regions which are functionally connected to each of the M1 seeds were displayed in Figure 2. When compared with HCs, ICCI patients demonstrated decreased FC in bilateral lingual gyrus (LG), left fusiform gyrus (FG), and right primary sensorimotor cortex (PSMC) when the seed was located in the left A4hf, as well as decreased FC in bilateral FG and left PSMC when the seed was located in the right A4hf. Moreover, ICCI patients showed decreased FC between the left A6cdl and right LG, left calcarine gyrus (CG), as well as decreased FC between the right A6cdl and right FG. In addition, decreased FC between the left A4ul and bilateral FG, right PSMC as well as decreased FC between the right A4ul and left LG, PSMC, right FG was also found in ICCI patients. Besides, when the seed was located in the left A4t, ICCI patients demonstrated decreased FC in right temporal pole (TP), LG and left FG (cluster-level FWE correction with *P* < 0.05) (Figure 3 and Supplementary Table 1).

However, when the seed was located in the bilateral A4tl, A6cvl or the right A4t, no statistically significant differences in FC were found.

### Correlation analyses between the functional connectivity value and clinical variables in incomplete cervical cord injury patients

In spearman correlation analyses, there were no correlations between the FC values in brain regions with significant group differences and the sensory scores, motor scores, or the injury duration of ICCI patients (*P* > 0.05).

## Discussion

Our study indicated that FC changes rather than structural changes occur in M1 subregions after ICCI. Each of the M1 subregions have diverse FC reorganizations, which may provide precise stimulation targets for the motor rehabilitation treatments, for example, TMS or tDCS. In addition, our findings also provide a preliminary theoretical basis for visual- and cognitive- related trainings [such as visual feedback, virtual reality (VR), and motor imagery (MI)].

### No morphological changes in the whole or each subregion of primary motor cortex in incomplete cervical cord injury patients

Previous studies have reported GMV changes in M1 following SCI (Freund et al., 2011, 2013; Hou et al., 2014b; Seif et al., 2018), which may be caused by direct or secondary WD (Nardone et al., 2018). The term WD refers to the progressive anterograde disintegration of axons and accompanying demyelination resulting from injury to the proximal axon or cell body (Buss et al., 2004). Previous studies suggest that WD can occur in the spinal cord of SCI subjects (Buss et al., 2004) and spreads along the corticospinal tract, eventually involving M1 and associated cerebral cortices (Freund et al., 2013; Nardone et al., 2018; Li et al., 2020). However, no significant structural difference either in the whole or in each subregion of M1 between groups was observed in our study. Our results were partly consistent with a few previous studies (Yoon et al., 2013; Villiger et al., 2015; Chen et al., 2017; Pan et al., 2017; Guo et al., 2021), which failed to find any GMV changes in the whole M1 after SCI. An early study found that the affected upward distance of WD was only about 6–7 mm, which can hardly involve M1 (Pallini et al., 1988). In addition, the continued activation of axotomized cells, as well as the extensive network of collaterals from pyramidal cells in M1 may contribute to prevent cell shrinkage, thereby preventing the volume atrophy in M1 after SCI (Chen et al., 2017; Nardone et al., 2018).

### Altered functional connectivity of primary motor cortex subregions in incomplete cervical cord injury patients

#### Decreased functional connectivity in sensorimotor related brain regions

After SCI, neural plasticity occurs at multiple levels, mainly in the cortical representation of sensory motor areas and these reorganizations may influence the prognosis of SCI patients (Athanasiou et al., 2017). In our study, compared with HCs, significantly decreased FC in contralateral PSMC of ICCI patients was found when the seed were located in bilateral A4hf or A4ul. PSMC is the core hub of sensorimotor function, which plays an important role in the motor planning, initiation and execution (Mohammed and Hollis, 2018). Our results were partly consistent with previous studies which found decreased interhemispheric FC between the bilateral M1 (Hou et al., 2014a; Athanasiou et al., 2017). These findings suggest possible decreased interhemispheric communication and collaboration, which may be due to the loss of synchronization between sensorimotor areas of the two cerebral hemispheres caused by the afferent/efferent imbalance after SCI (Kaushal et al., 2017). At present, taking the whole M1 as the stimulation target, TMS or tCDS, sometimes, is difficult to obtain satisfactory therapeutic effect (Tazoe and Perez, 2015; Kumru et al., 2016). Our study revealed the decreased FC between A4hf or A4ul and contralateral PSMC after SCI, suggesting that A4hf and A4ul may be used as the precise targets of TMS or tCDS, however, further research is still needed.

#### Decreased functional connectivity in visual related brain regions

In addition to the decreased FC between M1 and sensorimotor brain areas, when the seed was located in bilateral A4hf, A6cdl, A4ul or the left A4t, the ICCI patients showed decreased FC in visual related brain regions, including LG, FG, and CG. Our findings were partly consistent with the results of previous studies (Hawasli et al., 2017; Li et al., 2020), which used the whole M1 as ROI and found decreased FC between M1 and visual related regions in SCI patients. Besides processing visual imagery, LG is also in charge of making visual associations and episodic memory consolidation, as well as being involved in the network of regions associated with verbal declarative memory (Fusar-Poli et al., 2009). FG occupies the largest anatomical portion of the ventral temporal cortex and is considered crucial for processing high-level vision such as face perception, object recognition, and reading (Weiner and Zilles, 2016). CG is also located in the visual cortex and play a key role in the perception and processing of visual stimulation (Hu et al., 2021). Several studies have shown that visual and memory informations play an important role in regulating sensory and motor functions (Seidler and Carson, 2017; Li et al., 2020). Using additional visual information, patients would be able to better understand the orientation and displacements of the body in space (Walker et al., 2000). And during obstacle crossings, vision provides crucial information for identifying potential hazards in the environment, as well as for planning trajectories (Malik et al., 2017). Moreover, visual feedback (Houston et al., 2021) and VR (De Miguel-Rubio et al., 2020) have proven to be effective rehabilitation training methods, suggesting that the visual association cortex may play an important role in regulating sensory and motor functions, although the specific mechanism remains unclear (Li et al., 2020). Therefore, we speculated that the reduced sensory and motor function after ICCI might result in the decreased FC between the subregions of M1 and visual related regions. Visual related rehabilitation trainings, such as visual tactile feedback and VR, might contribute to the improvement of motor function after SCI by improving the decreased FC.

#### Decreased functional connectivity in cognitive related brain regions

When the seed was located in left A4t, we also found that ICCI patients showed reduced FC in the right TP, which is a critical area for the processing of information relevant to high-level cognition (Fan et al., 2014). Located in the anterior portion of temporal lobe, TP not only has been considered to be a structurally uniform area, but also has been proved as a functionally homogeneous region (Fan et al., 2014). Numerous studies have demonstrated that TP is an association cortex capable of multisensory integration and participates in various high-order cognitive functions, including the higher-order aspects of language, empathic behavior, memory, name and face recognition, emotion, and social cognition (Skipper et al., 2011; Fan et al., 2014). Previous studies found that cognitive processes play an important role in the early motor learning, adaptation, and regulation (Seidler and Carson, 2017). Meanwhile, some scholars also revealed that motor may affect cognitive ability by affecting brain activation and connections between brain regions (Kirk-Sanchez and McGough, 2014). Therefore, we speculate that the decrease FC between the left A4t and the right TP may be associated with the loss of sensorimotor function, which results in the depression and cognitive impairment after SCI (Williams and Murray, 2015). And cognitive training, such as MI (an active mental rehearsal of movements without physical execution) (Vidaurre et al., 2021), may be an effective treatment to improve motor function after SCI.

### Limitations

There are several limitations of our study. First, the sample size of this study was relatively small. Second, despite the fact that all of the patients in this study had incomplete cervical cord injuries, the degrees of injury varied, and future studies will focus on patients with more consistent injury degrees. Third, this study was a cross-sectional study. Further clinical studies are required to confirm the precise stimulation targets of TMS or tCDS and the effect of visual or cognitive related trainings on motor rehabilitation of SCI patients.

## Conclusion

Our observations suggested that the reorganization of M1 subregions occurs in function rather than structure after ICCI. Significantly decreased FC was observed not only in the sensory motor related brain areas, but also in the visual- and cognitive- related brain areas, which will help to not only accurately select the stimulation targets of motor rehabilitation therapy, but also provides a theoretical basis for visual and cognitive training (such as visual feedback, and motor imagery) to promote motor function.

## Data availability statement

The original contributions presented in this study are included in the article/Supplementary material, further inquiries can be directed to the corresponding author.

## Ethics statement

The studies involving human participants were reviewed and approved by the Ethics Committee of Xuanwu Hospital of Capital Medical University. Written informed consent to participate in this study was provided by the participants’ legal guardian/next of kin.

## Author contributions

LW and NC conceptualized the study. LW, WZ, BY, QC, XL, XC, and NC designed and conducted the experiments. YH, LC, JR, YY, and JL contributed to data collection. LW and WQ analyzed the data. LW wrote the manuscript. NC supervised the manuscript. All authors contributed to the study and approved the submitted version.

## Footnotes

1
https://www.physio-pedia.com/American_Spinal_Cord_Injury_Association_(ASIA)_Impairment_Scale

2
https://www.nitrc.org/projects/mricron/

3
http://www.neuro.uni-jena.de/cat12/CAT12-Manual.pdf

4
http://www.rfmri.org/dpabi

5
http://www.fil.ion.ucl.ac.uk/spm
