# Supplementary material for: Altered functional connectivity between primary motor cortex subregions and the whole brain in patients with incomplete cervical spinal cord injury
Source: Front Neurosci. 2022 Nov 3;16:996325. doi: 10.3389/fnins.2022.996325 (PMC9669417; doi:10.3389/fnins.2022.996325)
Supplement: Supplementary file 1 [file Table_1.docx]

Supplementary Material

# Table S1. Brain regions with decreased FC in M1 subregions of ICCI patients

| Subregions of M1 | Brain regions with decreaced FC | MNI coordinate | | | Cluster size (voxels） | P value | Peak T value |
| --- | --- | --- | --- | --- | --- | --- | --- |
|  |  | x | y | z |  |  |  |
| A4hf_L | LG_R | 30 | -48 | -6 | 211 | ＜0.001 | -5.09 |
|  | FG_L | -36 | -69 | -3 | 128 | 0.005 | -4.81 |
|  | LG_L | -18 | -81 | -9 | 101 | 0.016 | -3.96 |
|  | PSMC_R | 30 | -24 | 69 | 75 | 0.048 | -5.31 |
| A4hf_R | FG_R | 30 | -51 | -3 | 182 | 0.001 | -5.01 |
|  | FG_L | -36 | -69 | -3 | 206 | ＜0.001 | -5.03 |
|  | PSMC_L | -15 | -36 | 69 | 133 | 0.004 | -4.22 |
| A6cdl_L | LG_R | 30 | -48 | -6 | 174 | 0.001 | -5.33 |
|  | CG_L | -24 | -60 | 3 | 113 | 0.011 | -5.52 |
| A6cdl_R | FG_R | 33 | -54 | -3 | 109 | 0.012 | -5.24 |
| A4ul_L | FG_R | 30 | -51 | -3 | 397 | ＜0.001 | -5.98 |
|  | FG_L | -33 | -57 | -15 | 476 | ＜0.001 | -6.21 |
|  | PSMC_R | 27 | -24 | 69 | 176 | 0.001 | -7.08 |
| A4ul_R | LG_L | -21 | -51 | 0 | 136 | 0.004 | -4.50 |
|  | FG_R | 30 | -51 | -6 | 293 | ＜0.001 | -5.43 |
|  | PSMC_L | -51 | -18 | 54 | 148 | 0.003 | -4.96 |
| A4t_L | TP_R | 33 | 12 | -30 | 108 | 0.010 | -4.51 |
|  | LG_R | 30 | -54 | 0 | 552 | ＜0.001 | -5.76 |
|  | FG_L | -33 | -57 | -18 | 343 | ＜0.001 | -5.22 |

Note: FC: functional connectivity, M1: primary motor cortex, ICCI: incomplete cervical cord injury, MNI: Montreal Neurological Institute, LG: lingual gyrus, FG: fusiform gyrus, PSMC: primary sensorimotor cortex, CG: calcarine gyrus, TP: temporal pole, L: left, R: right.
